# LESSONS LEARNED FROM THE FIRST INTERVENTION STUDY ADDRESSING DEMENTIA IN THE LGBT COMMUNITY

**DOI:** 10.1093/geroni/igz038.1249

**Published:** 2019-11-08

**Authors:** Karen Fredriksen Goldsen, Karen Fredriksen-Goldsen, Linda Teri, Hyun-Jun Kim, Glenise McKenzie, David La Fazia, Charles Emlet, Ryan Petros

**Affiliations:** 1 University of Washington, Seattle, Washington, United States; 2 Oregon Health & Science University, Portland, Oregon, United States; 3 University of Washington, Tacoma, Washington, United States

## Abstract

The cognitive health needs of LGBT older adults have not been adequately addressed in mainstream clinical trials. Aging with Pride: IDEA (Innovations in Dementia Empowerment and Action), is an intervention designed to improve physical functioning and quality of life of LGBT adults with dementia and caregivers. We evaluate the processes and effectiveness of culturally-responsive recruitment approaches implemented in this study. A strong research-community partnership was necessary for the development and implementation of the intervention. LGBT participants with dementia made the first contact to research team as often as caregivers did and showed a higher rate of living alone and having a friend-based care network. The most common reason for ineligibility was not having a caregiver. Participants learned about the study via multiple venues including community events, newsletters, and social media. This study illustrates important new ways to sustain collaborations with disadvantaged communities and conduct a clinical trial with hard-to-reach participants.

